# A new species of *Zingiber* (Zingiberaceae) from Natma Taung National Park, Chin State, Myanmar

**DOI:** 10.3897/phytokeys.138.46719

**Published:** 2020-01-10

**Authors:** Ren Li, Law Shine, Wu Li, Shi-Shun Zhou

**Affiliations:** 1 Center for Integrative Conservation, Xishuangbanna Tropical Botanical Garden, Chinese Academy of Sciences, Menglun, China Center for Integrative Conservation, Xishuangbanna Tropical Botanical Garden, Chinese Academy of Sciences Menglun China; 2 Southeast Asia Biodiversity Research Institute, Chinese Academy of Sciences, Yezin, Nay Pyi Taw 05282, Myanmar Southeast Asia Biodiversity Research Institute, Chinese Academy of Sciences Nay Pyi Taw Myanmar; 3 University of Chinese Academy of Sciences, Beijing, China University of Chinese Academy of Sciences Beijing China; 4 Natma Taung National Park, Natural and Wildlife Conservation Division, Forest Department, Myanmar Natural and Wildlife Conservation Division Kanpletlet Myanmar; 5 College of Agriculture and Forestry, Pu’er University, Pu’er, Yunnan 66500, China Pu’er University Pu’er China

**Keywords:** *Zingiber* new species, Myanmar, Taxonomy, Zingiberaceae

## Abstract

*Zingiber natmataungense* S.S.Zhou & R.Li (Zingiberaceae), a new species from Natma Taung National Park, Chin State, Myanmar, is described and illustrated. The new species is morphologically similar to *Z. yunnanense*, but differs by: leaf blade abaxially light green, glabrous, ligule sparsely pubescent, ca. 2–3 mm, bracts glabrous; calyx white 20–21 × 3.2–3.5 mm, glabrous, apex obviously 3-toothed; corolla tube white, ca. 3.9–4.1 cm, labellum lateral lobes, ca. 1.5–1.7 × 0.6–0.7 cm; stamen with sparse pubescent, filament white, glabrous, 1–2 mm; anther connective appendage yellowish proximally, purplish distally; ovary white, sparsely white pubescent, epigynous glands, ca. 6–7 mm long, tapered, apex whorled, yellow. This new species is also similar to *Z. teres*, but has a different flower colour.

## Introduction

Zingiberaceae is a pantropical and subtropical family, but with most species distributed in South and Southeast Asia. Zingiberaceae consist of about 50 genera and 1300 species. There are about 100 to 150 species in *Zingiber*, out of which 42 occur in China (Wu and Larsen 2000). Plants of the genus *Zingiber* are widely used throughout the world as foods and as herbal remedies in various traditional healing systems because of their wide range of bioactivities (Sharifi-Rad et al. 2017). In the last decade, one new genus and several new species of Zingiberaceae have been described from Myanmar (Kress et al. 2010; Gowda et al. 2012; Ding et al. 2018; Tanaka and Aung 2019). Two new species of *Zingiber* were reported recently from the west and northwest of Myanmar (Aung et al. 2017; Tanaka and Aung 2017). Plant diversity in Myanmar has certainly been underestimated so far and there is an urgent need for both botanical exploration and plant conservation (Jin et al. 2018).

Since 2014, cooperation between the Ministry of Natural Resources and Environmental Conservation in Myanmar and the Chinese Academy of Sciences (CAS) has resulted in more than ten joint biodiversity investigations in northern and western Myanmar by researchers from the Forest Department of Myanmar and CAS institutions. During our investigations from October 2016 to July 2019, in Natma Taung National Park, Chin State, western Myanmar, a new species of *Zingiber* was discovered and is described as follows.

## Materials and methods

According to the published method (Stearn 1983), the morphological description of the new species was prepared from living plants and five dried herbarium specimens (HITBC: herbarium of Xishuangbanna Tropical Botanical Garden, the Chinese Academy of Science). Measurements were made using a vernier caliper. Herbarium and fresh specimens of *Zingiber yunnanense* (KUN: herbaria of Kunming Institute of Botany, the Chinese Academy of Science, Specimen number No.0833231 or No.0833232) and *Zingiber teres* (KUN, Specimen number No. 0833210) were also examined.

## Taxonomy

### Zingiber natmataungense

Taxon classificationPlantaeZingiberalesZingiberaceae

S.S.Zhou & R.Li sp. nov.

B72A33CD-6A5F-5039-AD48-49A1210A3F98

urn:lsid:ipni.org:names:77204198-1

Fig. 1

#### Diagnosis.

*Zingiber natmataungense* is similar to *Zingiber yunnanense* S. Q. Tong & X. Z. Liu (Tong and Liu 1991; Wu and Chen 1997), but differs from it by leaf blade abaxially light green, ligule sparse pubescent, bracts glabrous; calyx white and glabrous, apex obviously 3-toothed; lateral lobes, ca. 1.5–1.7 × 0.6–0.7 cm; stamen with sparse pubescence, filament white and glabrous, 1–2 mm; anther connective appendage yellowish proximally, purplish distally; ovary white with sparse white pubescence; epigynous glands tapering, yellow. This new species also shows some morphological affinities with *Z. teres* S. Q. Tong & Y. M. Xia (Tong and Xia 1987), but differs from it by corolla tube white, ca. 3.9–4.1 cm; central lobe white; lateral lobes white, 28–30 × 4–5 mm; labellum central lobe white, apex undulate and lobed, purplish-spotted at base, 28–29 × 16–18 mm. (Fig. 2).

**Figure 1.**
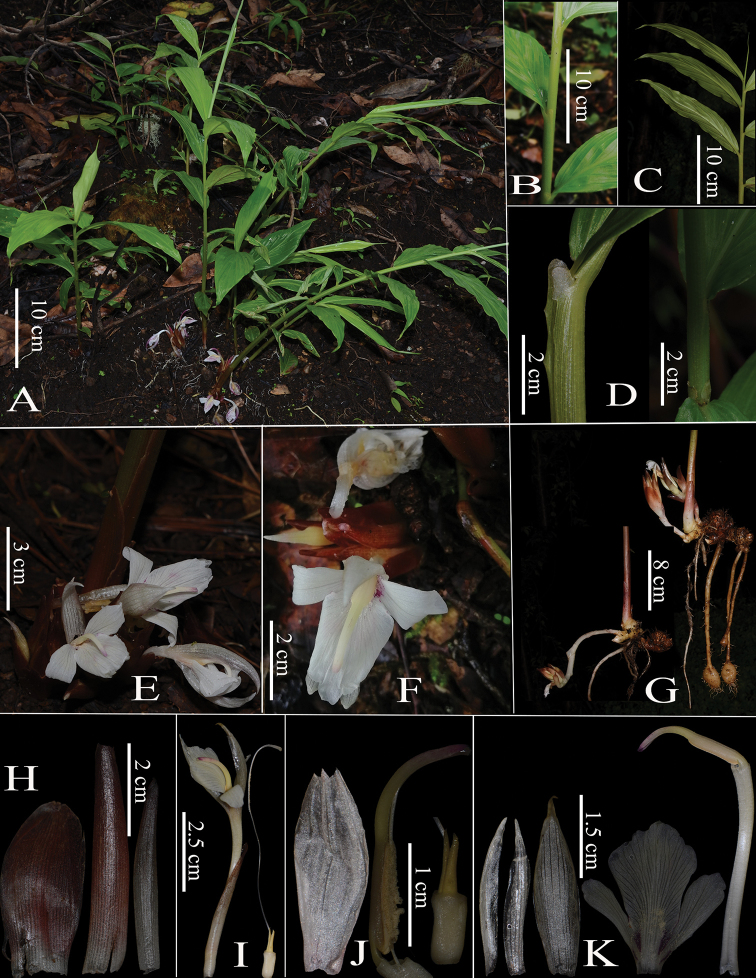
*Zingiber natmataungense* S.S.Zhou & R.Li, sp. nov. **A** habitat **B–D** pseudostem and detail of ligules **E** inflorescence **F** flower **G** inflorescence and rhizome **H** bract **I** flower and style **J** calyx and detail of ovary with epigynous glands and anther **K** dissection (from left): corolla lobes and labellum, floral tube with anther in side view.

**Figure 2.**
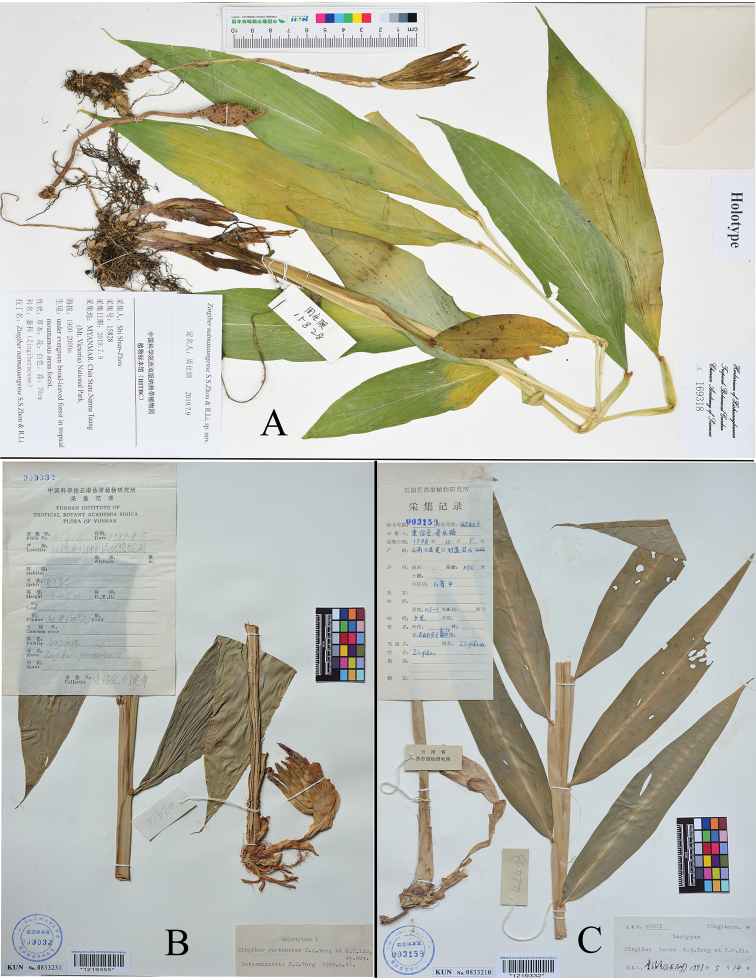
**A** Holotype of *Zingiber natmataungense* S.S.Zhou & R.Li, sp. nov (S.S. Zhou. 15828, HITBC Acc. No. 169318) **B** holotype of *Z. yunnanense* S.Q.Tong et X.Z.Liu (Tong, S.Q. & Liu, X.Z. 42412, KUN Acc. No. 0833231) **C** isotype of *Z. teres* S.Q.Tong et Y.M.Xia (Tong, S.Q. & Xia, Y.M. 42403, KUN Acc. No. 0833210).

#### Type.

Myanmar. Chin State. Natma Taung (Mt. Victoria) National Park, under evergreen broad-leaved forest in tropical montane forest, 1900–2000 m alt., 9 July 2019, Shi Shun-Zhou 15828 (holotype: HITBC!, Herb. Bar. Code No. 169318; isotype: RAF!).

#### Description.

Pseudostems 50–80 cm, base with purplish-red sheaths. Rhizome yellow, aromatic. Leaves subsessile, ligule 2-lobed, 2–3 mm, sparsely pubescent; leaf blade green, abaxially light green, lanceolate or narrowly, ca. 5–25 × 3–5 cm, glabrous, base cuneate, apex acuminate or caudate. Inflorescences radical, ellipsoid, ca. 5–6 × 2–3.5 cm, ellipsoid or narrow ellipsoid; peduncle embedded in ground, 3–16 cm; bracts glabrous, outer ones purple, elliptic, apex blunt, ca. 4–4.2 × 2–2.3 cm, inner ones purple, purplish at base, long ellipsoid or lanceolate, ca. 4.5–5.0 × 1.3–1.7 cm; bracteoles white, purplish-spotted at apex, white at base, tubular, 43–45 × 4.5–5 mm. Calyx white 20–21 × 3.2–3.5 mm, apex obviously 3-toothed, glabrous. Corolla tube white, glabrous, ca. 3.9–4.1 cm; central lobe white with apex caudate-acuminate, ca. 3.1–3.3 × 0.8–0.9 cm; lateral lobes with acuminate apex, 2.8–3.0 × 0.4–0.5 cm. Labellum white, glabrous, apex undulate and lobed, purplish plaque at base; central lobe obovate, ca. 2.8–2.9 × 1.6–1.8 cm; lateral ones oblanceolate, ca. 1.5–1.7 × 0.6–0.7 cm. Stamen with sparse pubescence, ca. 2.4–2.6 cm; filament white, glabrous,1–2 mm; anther yellowish, ca. 1–1.1 cm; connective appendage yellowish proximally, purplish distally, ca. 1.4–1.6 cm. Ovary white, sparsely white pubescent; style white, glabrous, stigma slightly thicker than style, white, ostiole front facing, ciliate. Epigynous glands 2, ca. 6–7 mm tapered, apex whorled, yellow. Fruit unknown.

#### Etymology.

The new species is named after Natma Taung National Park, Chin State, Myanmar, where it was discovered in a vast area of monsoon forest.

#### Phenology.

Flowering from July to August.

#### Distribution and habitat.

*Zingiber natmataungense* is only known from the type locality. It is a terrestrial plant in monsoon forest dominated by *Castanopsis tribuloides* (Smith) A. de Candolle (Fagaceae) and *Nyssa javanica* (Blume) Wangerin (Nyssaceae) and narrowly distributed from 1900 m to 2000 m alt. It has been used as a traditional medicine by local Chin people, who cover wounds with freshly crushed rhizomes and also apply it as a substitution for common ginger to treat coughs by drinking water in which it has been boiled.

#### Critical note.

The new species most resembles *Zingiber yunnanense* and *Z. teres*. Detailed morphological differences between the two species are given in Table 1.

**Table 1.** Diagnostic morphological characters of *Zingiber natmataungense*, *Z. yunnanense* and *Z. teres*.

Characters	*Zingiber natmataungense*	*Zingiber yunnanense*	*Zingiber teres*
Leaf blade	abaxially light green, glabrous	abaxially purplish-red on basal leaves, sparsely hairy	glabrous except sparsely puberulent along mid-vein abaxially
Ligule	sparsely pubescent, ca. 2–3 mm	densely pubescent, 4–7 mm	pubescent, 2–4 mm
Bracts	glabrous	slightly hairy	glabrous except red pubescent at acute or acuminate apex
Calyx	white 20–21 × 3.2–3.5 mm, glabrous, apex obscurely 3-toothed	white with red base and apex, ca. 10 mm, sparsely hairy, apex truncate	apex obscurely 3-toothed, 14–16 mm
Corolla tube	white, ca. 3.9–4.1 cm	white with red apex, ca. 3.7 cm	yellow, ca. 4–5 cm
Central lobe	White, 31–33 × 8–9 mm	red with slightly yellowish-green base, ca. 33 × 13 mm	yellow, 26–30 × 9–10 mm
Lateral lobes	White, 28–30 × 4–5 mm	red with slightly yellowish-green base, ca. 33 × 13 mm	yellow, 20–22 × 5–7 mm
Labellum central lobe	white, apex undulate and lobed, purplish- spotted at base, 28–29 × 16–18 mm	white with purple lines, elliptic, ca. 28 × 17 mm	purple with yellow stripes, apex acuminate, 18–20 × 11–13 mm
Labellum Lateral lobes	ca. 1.5–1.7 × 0.6–0.7 cm	ca. 0.7 × 0.45 cm	small
Stamen	sparsely pubescent,	glabrous	glabrous
Filament	white, glabrous, 1–2 mm	no filament	no filament
Anther connective appendage	yellowish proximally, purplish distally, 14–16 mm	purplish, 2-cleft ca. 15 mm	yellow proximally, purple distally, ca. 10 mm
Ovary	sparsely white pubescent	densely white pubescent	densely white pubescent
Epigynous glands	yellow, ca. 6–7 mm, tapered, apex whorled	white, ca. 5 mm, linear	white, ca. 4 mm, linear

## Supplementary Material

XML Treatment for Zingiber natmataungense
